# Acute Dehydration Impairs Performance and Physiological Responses in Highly Trained Judo Athletes

**DOI:** 10.3390/biology11060872

**Published:** 2022-06-06

**Authors:** Bayram Ceylan, Rafael L. Kons, Daniele Detanico, Jožef Šimenko

**Affiliations:** 1 Department of Coaching Education, Faculty of Sport Sciences, Kastamonu University, Kastamonu 37150, Türkiye; 2 Department of Physical Education, Federal University of Bahia, Salvador 40170-110, BA, Brazil; rafakons0310@gmail.com; 3 Biomechanics Laboratory, Center of Sports, Federal University of Santa Catarina, Florianópolis 88040-900, SC, Brazil; danieledetanico@gmail.com; 4 School of Life and Medical Sciences, University of Hertfordshire, Hatfield AL10 9EU, UK

**Keywords:** combat sports, hypohydration, judo performance, handgrip strength

## Abstract

**Simple Summary:**

This study investigated the effect of acute dehydration on judo-specific performance and physiological responses in highly trained judo athletes. Athletes performed judo-specific tests—such as maximal handgrip strength, dynamic and static judogi grip strength tests, and the Special Judo Fitness Test—in both dehydration and control conditions. The findings revealed that dehydration of 5% body mass caused impairment in dynamic and isometric strength in upper limbs and judo-specific performance, as well as elevated HR during the Special Judo Fitness Test.

**Abstract:**

Background: The present study investigated highly trained male judo athletes and how a 5% body mass dehydration affects their judo-specific performance and physiological responses. Methods: Nine highly trained international-level male judo athletes who are weight-cyclers voluntarily participated in the study. The study had a controlled crossover design in nature. Athletes completed three sessions, including a familiarisation session and two experimental sessions (dehydration (DEHY) and control (CON)) with judo-specific tests, including maximal handgrip strength test (HGS), judogi grip strength tests (JGST), and the Special Judo Fitness Test (SJFT). Results: Intergroup analysis revealed a significant increase in urine specific gravity (USG) and decreased body mass following DEHY condition compared to CON. Furthermore, significant decreases were determined in HGS, JGSTs, and a number of throws in the first and third series of SJFTs, as well as higher heart rate (HR) responses in the second and third series of SJFT and worse SJFT index in DEHY condition (*p* < 0.05). Conclusions: We concluded that 5% dehydration of body mass led to impairment in dynamic and isometric strength in upper limbs and in judo-specific performance, as well as elevated HR during the SJFT.

## 1. Introduction

Judo is an Olympic combat sport where athletes compete according to weight categories [1]. In this sense, weight cutting has been a common practice to fight in the desired weight category [2,3] to gain an advantage against their opponent who does not lose weight. However, the main concern about weight reduction is related to the amount of weight loss and the time spent doing it. To compete in a lighter weight class, combat sports athletes usually practice rapid weight loss (RWL), which is a reduction of approximately 2–10% of their body mass before the competition, mostly 2–3 days prior to weigh-in [3,4]. Previous studies have shown that some of the most frequently used methods for RWL are the fluid restriction or dehydration strategies, such as using sauna and plastic clothing [5]; or more aggressive methods, such as diuretics and laxatives [6]. It is known that dehydration may lead to a decrease in athletic skill and motor function to a great extent [7,8,9], increased heart rate due to the effort [10], renal impairment in severe cases [11], and adverse effects on neuromuscular and cognitive systems [12,13]. 

To date, some studies have investigated the effect of dehydration in combat sports. For example, Moore et al. [14] suggested that acute dehydration compromises muscle strength, work capacity, and glycolytic energy release in wrestlers. Barley et al. [15] also reported decreased muscular strength-endurance performance and increased fatigue following acute dehydration of 3.2% body mass in combat sports athletes. Moreover, Barley et al. [16] applied some physical performance tests to mixed martial arts athletes and indicated reduced performance 3 h and 24 h following 5% acute dehydration. However, although dehydration is very common among judo athletes from different competitive levels and categories [4,17,18,19], we have not come across any study investigating the effect of acute dehydration that is likely presented in judo competitions (significant to serious dehydration) [4,20] on judo-specific performance. Furthermore, much of the research investigated different weight loss methods in the laboratories, but they did not provide a clear implication related to the influence of acute dehydration on judo-specific performance [21,22,23,24].

In the literature, some markers have been used to estimate the dehydration level of an individual. A relevant biomarker to measure the magnitude of dehydration is the urine specific gravity (USG), which is one of the methods accepted by the National Athletic Trainers’ Association (NATA) [25]. It is known that judo athletes are exposed to constant weight loss using dehydration-related practices as judo competitions are spread throughout the year [1,20]. Moreover, there is clear evidence to suggest that judo athletes do not show optimal hydration status during training and competition despite rapid weight gain between weigh-in and competition [4,18,20,26]. However, there is no conclusive evidence about the effect of acute dehydration on judo-specific performance or understanding of the effects of the dehydration process. Thus, the results following manipulated hydration status via RWL on judo-specific performance may provide a more accurate response to the negative consequences of this method, which should be taken into account by the coaches who are the most effective people on athletes regarding RWL [27]. Considering these aspects, this study aimed to determine the effect of acute dehydration on physical performance (generic and judo-specific tests) and physiological responses (i.e., heart rate and blood lactate concentration) in highly trained judo athletes. We hypothesized that dehydration of 5% body mass would induce impairments in physical performance and physiological responses.

## 2. Materials and Methods

### 2.1. Study Design

**Figure 1:**
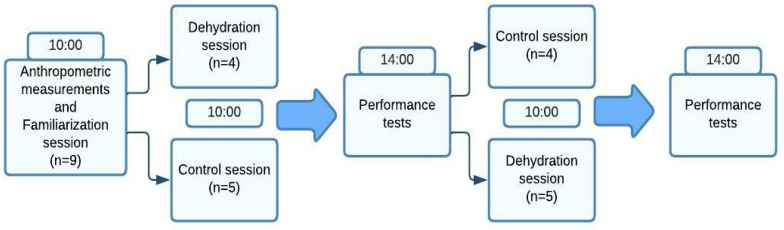
Study Design.

This study had a controlled crossover design in nature (Figure 1). Athletes completed three sessions, including a familiarisation session and two experimental sessions: control (CON) and dehydration (DEHY). The athletes completed the same series of performance tests in the same order during the familiarisation session as follows: maximal handgrip strength, maximal judogi grip strength tests (JGSTs), and the Special Judo Fitness Test (SJFT) interspersed with 10-min intervals. During the experimental sessions, athletes were randomly involved in either CON or DEHY protocol during the first experimental session and then they were crossed. The same researchers carried out the measurements at the same time of the day and under the same conditions, and separated by 7 days during which athletes refrained from any strenuous exercise. Athletes were given 1 h to refeed and rehydrate between dehydration/control protocols and performance measurements.

### 2.2. Subjects

Nine male highly trained international-level (participation in international competitions such as World/European Championships, Grand Slam/Prix, or continental cups) judo athletes with the following characteristics (mean and standard deviation): 19 ± 2 years of age, 71.4 ± 9.2 kg of body mass, 172.1 ± 6.0 cm of height, 12.8 ± 8.4 body fat percentage, 24.0 ± 2.2 body mass index, 61.5 ± 7.3 muscle mass, and 9.5 ± 1.5 years of judo practise (having at least first DAN) voluntarily participated in this study. None of the athletes were smokers, received any medical treatment, or had any dysfunction related to respiratory and circulatory systems and they were also supposed to be weight-cyclers. Being considered a weight cycler meant the athletes have resorted to rapid weight loss at least 5 times per year for the last 2 years. All athletes were informed about the nature of the study and related risks in detail. Written informed consent form was obtained from the athletes prior to the collection of data. The local Clinical Research Ethics Committee provided ethical approval of the study. 

### 2.3. Dehydration and Control Protocols

During dehydration, athletes wore a sauna suit (Mizuno, Japan) while cycling on a Monark cycle ergometer (Monark 928E, Vansbro, Sweden) at an exercise intensity of 60 W and 50 RPM in an environment that was set to 40 °C and 40% relative humidity for 3 h. Athletes were required to receive minimal fluid intake. In addition, a cycling activity was chosen to avoid possible eccentric muscle damage and any additional effects from the submaximal exercise on performance, as suggested by O’Reilly, et al. [28]. The cycle ergometer was adjusted for each athlete. In the final 5 min of every 25 min period of the DEHY condition, measurements of weight and USG were carried out. If an athlete was about to reach the weight loss of 5% body mass before the 3 h period, then less clothing was worn to minimise weight loss before the determined time. If athletes were over the 5% dehydration, then they consumed a small volume of water (150 mL) to achieve 5% dehydration. A 60 W workload was preferred during both protocols in order to increase core temperature and trigger sweating but also minimise muscle glycogen depletion, as glycogen depletion was associated with a decrease in performance [23]. The CON condition involved 3 h of exercise at 60 W under thermoneutral conditions without a sauna suit and with fluid intake.

### 2.4. Body Composition

A bioelectrical impedance (BIA) device (Tanita, BC-545, Tokyo, Japan), that uses a dual-frequency method (50 kHz/6.25 kHz), was used to assess athletes’ body composition. Measurements were performed according to manufacturer guidelines barefooted and in a standing position with legs and thighs apart (not touching). Before every measurement, skin and the electrodes were pre-cleared and dried. Additionally, the participants were asked [29]: (1) to abstain from large meals after 9:00 p.m. the evening before the test, and on the day of the measurement, they neither ate nor drank before the end of the procedure; (2) not to consume alcohol 48 h before the measurement; (3) to be in the standing position for at least 5 min before the test to redistribute the tissue fluids; (4) to position hands 15 cm laterally from the body, so they were not touching the torso. The BC-545 test–retest, reliability, and accuracy were previously assessed, with an interclass correlation (ICC) of 0.99. Additionally, the correlations with the reference measure (dual-energy X-ray absorptiometry—DXA) were shown to be significant at r  >  0.9 [29]. The following variables were obtained from the test: body mass, body fat percentage, muscle mass, and body fat percentage. Body mass index (BMI) was calculated from height and body mass using Quetelet’s equation [30]: BMI = body mass (kg)/height^2^ (m^2^). 

### 2.5. Maximal Handgrip Strength Test

The handgrip strength was measured in a standing position three times on each side alternately with 1-min intervals between attempts with a handgrip dynamometer (Takei, Japan) in both CON and DEHY conditions. Athletes were instructed to generate the maximum force during 3–5 s. Their tested arm was fully extended in the elbow joint and with self-selected wrist and leg positions. The best result for each side was used for further analysis. The reliability (ICC) and the typical error of measurement (TE) of maximal handgrip strength were assessed in the previous study [31], demonstrated good reliability values for dominant and non-dominant limbs (ICC = 0.96 and 0.97, respectively), and typical error was considered lower (TE = 5.96 and 4.62, respectively).

### 2.6. Judogi Grip Strength Test

Ten minutes following the maximal handgrip strength test, athletes performed a familiarisation involving the grip on the *judogi* sleeve and performed at least three dynamic repetitions and one isometric trial on the *judogi* suspended on the bar 72 h before the official assessment. Both dynamic and isometric versions of JGST were performed. The dynamic evaluation consisted of holding the *judogi* rolled around the bar with the elbow joint at maximal extension and performing elbow flexion, moving the chin above the line of the hands. Athletes were asked to perform the maximal number of repetitions from a fully extended to a fully flexed elbow position as many times as possible. After a 10-min interval, athletes performed the isometric test, which consists of sustaining the initial position (elbow fully flexed) for the maximal possible time. The chronometer was stopped when the athlete could no longer maintain the original position. The reliability of the JGST has been assessed in a previous study, presenting an intraclass correlation coefficient (ICC) higher than 0.98 for both tests [32] and the limits of agreement were 0.3 repetitions for dynamics and 2.3 s for isometric mode [33].

### 2.7. Special Judo Fitness Test

Following a 10-min recovery period after JGST, athletes performed a 5-min standardised particular warm-up, including jogging, judo falling techniques (*ukemi*), and repetitive throwing techniques without falling (*uchi-komi*). Subsequently, two judokas (*uke*) with similar height and body weight to the executor (*tori)* were positioned at a distance of 6 m from each other, while the *tori* was positioned 3 m from each *uke*. The test comprised of three periods—15 s (A), 30 s (B), and 30 s (C)—with 10-s intervals between them [34]. The *tori* threw the opponents with a hand technique (*ippon-seoi-nage*) as many times as possible. The final test performance of the *tori* was classified according to the total throws completed during all three periods (A + B + C). The total number of throws in combination with the heart rate (HR), which was measured immediately after the test and after 1 min (SEEGO RealTrack, Almeria, Spain), was used to calculate the index using the equation

$$\mathrm{Index}\;(\mathrm{bpm}\cdot {\mathrm{throws}}^{-1})=\frac{\mathrm{final}\;\mathrm{HR}\;(\mathrm{bpm})+\mathrm{HR}\;\mathrm{at}\;1\;\min\;\mathrm{after}\;\mathrm{the}\;\mathrm{test}\;\left(\mathrm{bpm}\right)}{\mathrm{Number}\;\mathrm{of}\;\mathrm{throws}}$$

Reliability values (ICC) of 0.73 and 0.88 for the number of throws and index have been previously reported, and TE was 2.58% and 4.58%, respectively [35].

### 2.8. Physiological Responses

Heart rate (HR) was monitored by a heart rate monitor (SEEGO RealTrack, Almeria, Spain) during the whole experimental process and recorded at rest, after the CON or DEHY protocols, during SJFT, and 1 min after the test. A lactate device (Edge Blood Lactate Monitoring System, ApexBio Inc., Taipei City, Taiwan) was used by the same experienced researcher to measure blood lactate (LA). LA was recorded at rest after the CON or DEHY protocols and 1 min after the test. The site was cleaned before each measurement with alcohol and dried with cotton, obtaining a 0.3 µL blood sample from the fingertip of the middle finger.

### 2.9. Hydration Assessment

A urine sample was taken from the participants before, at the last 5 min of every 25 min period during the dehydration condition, and after both experimental and control protocols. A digital refractometer (ATAGO, PAL-10S, Tokyo, Japan) was used to analyse the urine, initially placed in sterilised plastic cups and immediately disposed of after the analysis [20]. The NATA Position Statement cut-offs (≤1.020 g/mL euhydrated, ≥1.020 g/mL dehydrated) were used to classify athletes’ hydration status [36].

### 2.10. Statistical Analysis

The descriptive data are reported as means and standard deviations. The Shapiro–Wilk test and descriptive methods including skewness and kurtosis coefficients [37] were used to verify the normality of the data. The difference in USG and body mass were determined with repeated measures ANOVA (2 × 2). Paired *t*-test for dependent samples was used to identify the difference in performance tests between CON and DEHY conditions. Cohen’s *d* was used for effect size, classified as 0.0–0.25 trivial 0.21–0.65 small; 0.61–1.2 moderate; 1.21–2.0 large; and 2.1–4.0 5 very large for two dependent samples [38]. The 0.05 significance level was set for all analyses conducted using JASP software (version 0.11.1, JASP team, University of Amsterdam, Netherlands).

## 3. Results

Table 1 presents hydration, body mass changes, and LA and HR responses in the CON and DEHY conditions. Dehydration was obvious, especially considering the significant increase in USG values (i.e., increased concentration of solutes in the urine), and decrease in body mass. However, LA and HR did not change regardless of the condition.

Table 2 presents the judo-specific test performance of the athletes in the CON and DEHY conditions. There were lower values in the strength tests performance in the upper limbs (HGS and JGST) in the dehydration condition compared to control.

**Figure 2:**
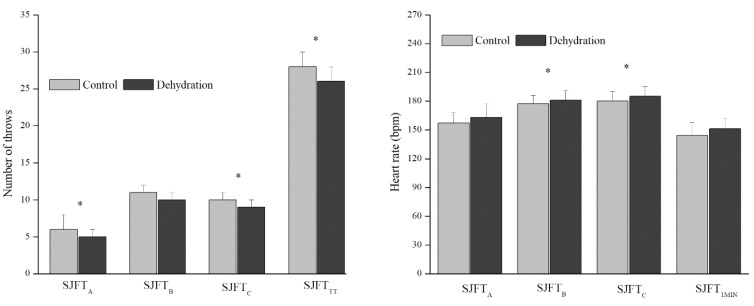
SJFT performance (number of throws) and heart rate in the control and dehydration conditions. A = First set (15 s), B = Second set (30 s) and C = Third set (30 s); TT = Total throw; * *p* < 0.05.

Figure 2 shows the SJFT performance and heart rate throughout the test in the CON and DEHY conditions. The number of throws was lower in the DEHY condition compared to CON in the first (8% delta change) and third series (7.9% delta change), while the heart rate was higher in the DEHY condition than CON in the second and third series. The SJFT index was higher (worse performance) in the DEHY condition (12.90 ± 1.16 bpm·throws^−1^) compared to CON (11.67 ± 0.94 bpm·throws^−1^, *p* < 0.001) with −1.3% delta change. The blood lactate concentration obtained 1 min after the SJFT did not show a difference between the conditions (control: 14.92 ± 3.57 mmol·L^−1^, dehydration: 17.01 ± 3.8 mmol·L^−1^, *p* = 0.119).

## 4. Discussion

The study aimed to analyse the effect of acute dehydration on physical performance and physiological responses in highly trained judo athletes. To the best of our knowledge, this study is the first to investigate the acute effect of dehydration on performance in judo athletes with sport-specific tests. The main findings of our study were: (1) there was a decrement in HGS and judo-specific tests (JGST and SJFT) after acute dehydration; and (2) there was an increase in HR during SJFT in the DEHY condition, but LA did not differ between the conditions. Therefore, we accept the hypothesis that the 5% body mass dehydration induced impairments in physical performance and led to increased cardiovascular load during the SJFT.

It was confirmed that 5% dehydration could lead to an increase in USG and a decrease in body mass. A few studies have investigated the hydration status of judo athletes and showed similar values to the baseline of our study. Ceylan, Barley and Balci [4] investigated the hydration status of elite judo athletes during a competition period and the USG values were 1.023 ± 0.002 g·mL^−1^ for a week before the competition 1.030 ± 0.001 g·mL^−1^ at the official weigh-in and 1.017 ± 0.007 g·mL^−1^ for 24 h post-match, respectively. Another study conducted by Stefanovsky, Clarys, Cierna and Matejova [26] monitored the hydration status of young judo athletes during an off-season training camp and indicated a high level of dehydration with a mean value higher than 1.020 g·mL^−1^ during the first three measurements and minimal dehydration levels for the last two days of the camp. In addition, Ceylan and Balci [20] reported a high level of dehydration in judo athletes with values of 1.027 ± 0.005 g·mL^−1^ for pre-weigh-in and 1.025 ± 0.005 g·mL^−1^ for pre-match (higher than our findings). Ceylan, Baydil and Aydos [18] also indicated that judo athletes presented a higher level of dehydration compared to wrestlers despite 15 vs. 2-h recovery period the following weigh-in. All of the abovementioned studies highlighted that judo athletes generally present a high level of dehydration, which made our findings important in terms of demonstrating the effect of dehydration on judo-specific performance. 

Dehydration may affect neural, cognitive, and metabolic responses in one, some, or all components [12]. For example, more significant dehydration decreased athletic skill and motor function to a great extent [7,8]. In addition, it was highlighted that cardiovascular impairment results from dehydration according to a study by Cheuvront, Carter and Sawka [10]. Furthermore, it has been reported that muscle membrane excitability [39] and the neuromuscular system [13] have been negatively affected by the reduction in body water. Therefore, athletes may be exposed to a greater physiological strain when they become dehydrated. In the light of the abovementioned information, it was found that when athletes were exposed to dehydration, they presented worse performance during upper limb strength test (HGS) and judo-specific tests (JGST and SJFT), indicating that strength-related abilities—as well as tasks that require aerobic and anaerobic sources (e.g., SJFT) [38]—are impaired with 5% dehydration. 

Contrary to our findings, Artioli, et al. [21] observed no effects of 5% acute weight loss with 4-h recovery on judo-specific performance; however, the authors did not use dehydration-induced weight loss methods and nor did they control the hydration status of the athletes. Moreover, Mendes, et al. [40] verified that chronic weight cycling does not protect combat sports athletes from the negative impact of RWL on performance, which is an unresolved issue. Our findings also showed increased HR measured throughout the SJFT, indicating high cardiovascular demand in DEHY conditions. This can be explained by the adverse effect of dehydration on the cardiovascular system with declined stroke volume, cardiac output, and increased heart rate [41]. 

Finally, the current study presents some limitations, such as the small sample size; however, considering the main variables that showed a significant difference, the current sample size allowed enough power (>0.8) to avoid any Type II statistical error. Besides, measurements were carried out 1 h after the DEHY and CON protocols in contrast to 15 h of recovery between official weigh-in and competition. However, judo athletes are known to present minimal/significant dehydration before competition despite 15 h of recovery [4,19]. This study also showed some strengths, as it allowed us to directly identify with judo-specific tests that acute dehydration methods provoke impairments in dynamic and isometric strength in the upper limbs and on judo-specific performance.

## 5. Conclusions

We concluded that 5% dehydration of body mass induced a decrease in performance of judo-specific tests, as well as physiological and strength parameters in highly trained judo athletes. Worse performance in judo-specific tests occurred during exercise in the thermoneutral environment when athletes were dehydrated. Additionally, there is further potential for decrements in performance when exercising indoors with judo uniforms. Therefore, judo coaches, athletes, and athletic personnel should pay attention to the impact of dehydration on judo-specific activity. They should be encouraged to regularly and closely monitor hydration status from day to day to optimise the performance and recovery of the athletes. Moreover, the practice of RWL using dehydration-induced methods must be avoided and, if possible, it should be banned due to the harmful effects on athletes’ performance and health.

## Figures and Tables

**Table 1:** Hydration, body mass, LA, and HR changes in the CON and DEHY conditions.

	Control	Dehydration	Factors		
	Mean ± SD	Mean ± SD	Intervention (DEHY and CONT)	Measurement Times ^#^	Intervention × Time
USG_pre_ (g·mL^−1^)	1.013 ± 0.003	1.014 ± 0.003	60.23 *	64.03 *	70.84 *
USG_post_ (g·mL^−1^)	1.018 ± 0.003	1.029 ± 0.002			
Body mass_pre_ (kg)	71.2 ± 9.3	71.2 ± 9.3	459.19 *	538.11 *	394.24 *
Body mass_post_ (kg)	71.2 ± 9.3	67.6 ± 8.9			
LA_rest_ (mmol·L^−1^)	1.5 ± 0.4	1.5 ±0.4	9.12	169.12 *	2.29
LA_pre-exp_ (mmol·L^−1^)	2.6 ± 0.7	3.0 ± 0.9			
LA_1minpost_ (mmol·L^−1^)	14.9 ± 3.5	17.0 ± 3.8			
HR_rest_ (bpm)	73.8 ± 7.9	74.7 ± 6.6	5.97 ^#^	469.56 *	1.83
HR_pre-exp_ (bpm)	92.3 ± 10.1	97.5 ±10.0			
HR_1minpost_ (bpm)	144.2 ± 14.2	150.7 ± 10.6			

* *p* < 0.001, ^#^ *p* < 0.05, ^#^ pre and post experiment; rest, pre, and post experiment.

**Table 2:** Physical performance and physiological responses in the CON and DEHY conditions.

	CON	DEHY			95%CI Cohen’s *d*		
	Mean ± SD	Mean ± SD	*p*	Delta Change (%)	Cohen’s *d*	Lower	Upper
HGS_RIGHT_ (kgf)	49.5 ± 4.5	47.6 ± 3.8	0.004	3.8	1.34	0.40	2.23
HGS_LEFT_ (kgf)	47.8 ± 4.6	45.7 ± 5.4	0.037	4.4	0.83	0.04	1.58
JGST_ISO_ (s)	53.6 ± 11.5	45.8 ± 10.6	<0.001	14.6	1.88	0.74	2.97
JGST_DIN_ (rep)	21 ± 3	16 ± 4	0.005	23.8	1.28	0.36	2.16

HGS = handgrip strength test; JGST = judogi grip strength test; ISO = isometric; DIN = dynamic.

## Data Availability

The datasets used and/or analysed during the current study are available from the corresponding author on reasonable request.
